# Standardization of the methods and reference materials used to assess virus content in varicella vaccines

**DOI:** 10.1186/s12985-015-0333-1

**Published:** 2015-07-05

**Authors:** JiYoung Hong, Ho Jung Oh, Naery Lee, Do-Keun Kim, Heui-Seong Yoon, Yeon-Tae Kim, Seokkee Chang, Jae-Hak Park, Hyejoo Chung

**Affiliations:** Vaccines division, National Institute of Food & Drug Safety Evaluation, Ministry of Food and Drug Safety, Cheongju-si, 363-700 Republic of Korea; Department of Laboratory Animal Medicine, College of Veterinary Medicine, Seoul National University, Seoul, 151-742 Republic of Korea; ᅟ, 187 Osongsaengmyeoung2(i)-ro, Osong-eup, Heungdoek-gu, Cheongju-si, Chungcheongbuk-do 363-700 Republic of Korea

**Keywords:** Varicella, Vaccine, Virus content, National Center for Lot Release, Korean reference material

## Abstract

**Background:**

In Korea, every vaccine lot is tested by the National Center for Lot Release (NCLR) in accordance with the national lot release procedures to ensure the safety and efficacy of vaccines. These quality tests examine the virus content in varicella vaccines *via* plaque assays (either the agar overlay method [AOM] or plaque staining method [PSM]), according to the procedures suggested by the Korean Reference Material for the Varicella Vaccine (KRMVV) or the manufacturer’s standard in-house protocol.

**Aim:**

To standardize the virus content tests, viral titers in the KRMVV were measured using the PSM at four participating laboratories in a collaborative study. With the aim of developing a standardized method using the KRMVV as a positive control, we compared the ability of the two test methods, AOM and PSM, to accurately and reproducibly determine the virus content of two commercial varicella vaccines.

**Results:**

The results showed that the standardized method (PSM) was more suitable for quality control analysis of the varicella vaccine.

**Conclusion:**

Use of a standardized method (PSM) according to the Korean reference material will improve the reliability and objectivity of lot release testing.

## Introduction

Many studies have investigated whether a single infection with the varicella virus followed by recovery can confer permanent immunity to all varicella strains [1–3]. In 1974, Takahashi *et al*. developed a live attenuated varicella vaccine by isolating the Oka strain from patient specimens [4]. The vaccines currently used throughout the world have been developed from several different strains [5]. In Korea, selective varicella vaccination was approved and initiated in 1988, and varicella has been included in the national immunization program since May 2005. Varicella is designated as a secondary legal communicable disease [6]. Currently, varicella vaccines originating from the Oka and MAV/06 strains are approved for use in Korea [5, 7]. The varicella vaccines used in Korea must contain a viral titer of at least 1000 log_10_PFU/ dose. Therefore, the virus content is determined before use. The virus is diluted to within the range of 10^-2^ to 10^-3^, and virus-sensitive cells are then inoculated with the diluted virus and cultured to form plaques. Viable cells are stained with a dye, and the number of plaques formed is counted to calculate the virus content [8, 9]. Two virus content assays utilized for national lot release testing (Korean Minimum Requirement for Biological Products, Revision 6) use two different reference materials: Korean reference material (National Institute of Food & Drug Safety Evaluation, code no. 08/027) is used for the AOM, and an in-house standard protocol provided by the manufacturer is used for the PSM. As such, when using these assays to test varicella vaccine lots, virus content is obtained as either an absolute titer or a relative titer. Therefore, to improve the reliability and objectivity of lot release testing, we aimed to standardize the testing method and reference material used for determining virus content.

## Materials and methods

### Korean reference material for the varicella vaccine

The Korean reference material that is currently being used for the varicella vaccine was consigned to the Green Cross Corp. (Korea), first in 2002 (code: 02/006) and then again in 2009 (code: 08/027), and the MAV/06 strain (from a 33-month-old boy with chickenpox in1989 in Seoul, Korea) was manufactured for use as a standard for the AOM [10]. The Korean reference material used in this study (code: 08/027), was established by a collaborative study (assigned titer 4.26 log_10_PFU/ dose) *via* AOM [11]. The Korean reference material was stored at −70 °C, and annual monitoring was conducted to determine long-term stability.

### Sample vaccines

The commercially available products Suduvax (Green Cross Corp., Korea), produced from a MAV/06 virus strain, and Varilrix (GlaxoSmithKline, Belgium), produced from an Oka virus strain, were used as the test vaccine samples. Each vaccine sample used in the test was from the same lot that had passed a lot release test using national lot release procedures.

### Cells and methods

We used the same cell line, MRC-5 (pd19, Cat No. 5072101; European Collection of Cell Cultures, ECACC), for both the AOM and the PSM. MRC-5 cells were used within 30 population doubling levels (PDLs), and were maintained at 37 °C and 5 % CO_2_ in T175 flasks containing minimum essential medium (MEM; Gibco) supplemented with HEPES (Gibco), 2 mM L-glutamine (Gibco), antibiotics, and 10 % fetal bovine serum (FBS; Gibco). Cells were seeded at a density of 0.8–1 × 10^5^ cells/mL for 2 days before infection. In the AOM, MRC-5 cells were seeded in 6-well plates at a density of 2.4–3 × 10^5^ cells/well, and were incubated for 2 days at 37 °C and 5 % CO_2_. A monolayer of cells was inoculated with dilute virus samples, and was allowed to be infected for 90 min. The agar overlay medium contained MEM, 10 % FBS, 1 % penicillin (10,000 U/mL)/streptomycin (10 mg/mL) (Gibco), and 0.8 % (w/v) agarose (Lonza). The agar overlay medium was prepared by first heating the agar solution at 121 °C for 15 min. The solution was allowed to cool down to 40 °C prior to the addition of FBS and antibiotics. Following virus infection the agar overlay medium (primary overlay) was added to the wells and allowed to solidify at room temperature. On the fifth day of incubation, a secondary overlay of the same agar medium was added. Three days after the addition of the secondary overlay, a third overlay solution containing 0.33 % (w/v) neutral-red staining solution (Amresco) was added, and the number of plaques was then measured as the CPE.

In the liquid medium-based PSM, MRC-5 cells were seeded in 6-well plates at a density of 3 × 10^5^ cells/well, and incubated for 2 days at 36 °C and 3 % CO_2_. A diluted virus sample was inoculated into a monolayer of cells and was allowed to be absorbed for 90 min. Cells were incubated in MEM containing 50 μg/mL neomycin (Sigma), 7.5 % sodium bicarbonate solution (Gibco), and 2 % FBS (Gibco). After absorption, liquid medium was added. After 7 days of incubation, the liquid medium was removed, and cells were stained with 3 mL of Coomassie blue stain (Ethanol and Coomassie Brilliant Blue; Bio-Rad) for 30 min to 3 h. Finally, the number of plaques formed in the plate was counted over a light box.

### Collaborative study

The collaborative study was a 2-year project conducted from 2012 to 2013. In the first year, four laboratories, including the National Center for Lot Release, Green Cross Corp. (Korea), SK Chemicals (Korea), and Korea Vaccine Co. (Korea), participated in the collaborative study. PSM was used to calculate the assigned titer of the Korean reference material (code: 08/027), which had been previously determined using the AOM. In the second year, we determined the titer of varicella vaccines that were previously assessed in lot release testing using both a standardized PSM with the Korean reference material (code: 08/027) as a positive control, and the AOM. The results of the two testing methods were then compared. In 2012, the titer of the Korean reference material was measured 10 times (in triplicate) using the PSM at laboratories A, B, and D, and 11 times at laboratory C. Analyses were conducted on the results from the 33 tests performed at laboratory C as well as the 30 tests performed at each of the other three laboratories. In the second year (2013), with an aim to standardize the virus content assay used in lot release testing of the commercial varicella vaccine (Suduvax [Green Cross Corp., Korea] and Varilrix [GlaxoSmithKline, Belgium]), the viral titer of each vaccine was tested using a standardized PSM with Korean reference material as a positive control, and with the AOM. The results of the two testing methods were compared. The same laboratories that participated in the first year of the project continued to work as collaborators. In the present collaborative testing, each participating institution used the same method for AOM. However, in the case of the PSM, protocols at participating institutions were newly established. Nevertheless, training and SOP transfer from the present regulatory agencies were performed before the institutions performed the experiments independently.

### Statistical analysis

The SAS system version 9.2 was used to analyze the all the data collected.

## Results

To confirm the technical proficiency of the test laboratories, z-score analysis was performed. The median titer for each test organization and the normalized interquartile range (IQR) were used to calculate a robust z-score to reduce the effects of limit values on the entire statistic. In our collaborative study (2012), the robust z-score was within ± 1 in all laboratories (A = 0.80 (mean titer = 4.63 log_10_PFU/ dose), B = −0.96 (mean titer = 4.57 log_10_PFU/ dose), C = 0.47 (mean titer = 4.36 log_10_PFU/ dose), and D = −0.47 (mean titer = 4.43 log_10_PFU/ dose)); thus, the proficiencies of the test laboratories were considered to be sufficient (Table 1). Using the statistical testing methods described above, significant differences were found between the results of tests performed at the different laboratories. However, the results showed a good inter-laboratory variability (GCV =2.80 %) and the final titer of the Korean reference material was calculated by combining the estimated titers from the four laboratories.

**Table 1:** Results of the collaborative study using the national standard (National Institute of Food & Drug Safety Evaluation, code no. 08/027). The virus content test (by plaque staining method) results from the 4 participating laboratories (A, B, C, and D) were compared

Laboratory^*a*^	Robust Z score^*b*^	*p*-value^*^	Multiple Comparison^*c*^					
			A *vs*. B	A *vs*. C	A *vs*. D	B *vs*. C	B *vs*. D	C *vs*. D
A	0.8	<.0001	0.1364	<.0001	<.0001	<.0001	<.0001	0.0063
B	−0.96							
C	0.47							
D	−0.47							

^*a*^Laboratory: one of the participating laboratories, the National Center for Lot Release, Green Cross Corp. (Korea), SK Chemicals (Korea), and Korea Vaccine Co. (Korea) (in randomized order)

^*b*^Robust Z score: unlike the normal Z score, a robust Z score is calculated using the median and absolute deviation from median instead of the mean and standard deviation. When the robust z-score was < |2|, the proficiency of the test laboratories was considered satisfactory

^*c*^Multiple Comparison: p-value adjusted by Bonferroni method

^*^ *P*-value: ANOVA test

The overall titer was estimated by obtaining the geometric mean titer (GMT) with the non-weighted geometric mean using the GMT of each laboratory. The final GMT was 4.50 log_10_ PFU/ dose, and the geometric coefficient of variation (GCV) was 2.80 %, indicating that the measurements obtained by the different laboratories were similar. No difference was observed between the arithmetic mean and the arithmetic coefficient of variation (ACV).

In the second year (2013), the Korean reference material and vaccine samples were tested at each laboratory (Table 2). To confirm the reproducibility of the test results for the Korean reference material (code: 08/027) and vaccine samples, a robust z-score was calculated for the PSM and AOM at each concentration. The robust z-score was less than |2| for the two virus content tests and laboratories, indicating a satisfactory proficiency data not shown). In order to test the difference of potencies between the test agencies, One-way ANOVA analysis was performed. Potency of live varicella virus vaccine was calculated based on more than 30 repetitive tests at each institution. Each repeat was regarded as one independent experiment, and significant differences between test agencies were found (*p* < .0001). When multiple comparisons were performed by application of the Bonferroni method as a post-hoc comparison, every combination of institutions except A laboratory and B laboratory showed significant differences between institutions. However, geometric coefficients of variation (GCV) between test agencies were 1.09 % for A laboratory, 3.45 % for B laboratory, 0.98 % for C laboratory, and 0.37 % for D laboratory. This indicates that variations between test agencies were less than 5 %, confirming high reproducibility of the assay.

**Table 2:** Results of the collaborative study using the vaccine sample (Suduvax (Green Cross Corp., Korea) and Varilrix (GlaxoSmithKline, Belgium)). The results of the collaborative study by method were compared to the virus content (titer) of the vaccine (titer unit: log_10_PFU/ dose)

Vaccine	Laboratory^*a*^	Method	Result		
			GMT^*b*^	95 % C.I.^*c*^	*P*-value^*^
Suduvax	A	PSM^*d*^	3.53	3.49, 3.57	0.8661
		AOM^*e*^	3.54	3.50, 3.58	
	B	PSM^*d*^	4.01	3.98, 4.05	<.0001
		AOM^*e*^	3.28	3.25, 3.32	
	C	PSM^*d*^	3.74	3.67, 3.81	0.4627
		AOM^*e*^	3.70	3.64, 3.78	
	D	PSM^*d*^	3.68	3.66, 3.70	0.3658
		AOM^*e*^	3.70	3.68, 3.71	
Varilrix	A	PSM^*d*^	3.86	3.82, 3.91	0.5064
		AOM^*e*^	3.88	3.84, 3.93	
	B	PSM^*d*^	4.17	4.14, 4.20	<.0001
		AOM^*e*^	3.46	3.43, 3.49	
	C	PSM^*d*^	3.75	3.68, 3.82	<.0001
		AOM^*e*^	4.02	3.96, 4.10	
	D	PSM^*d*^	3.70	3.68, 3.72	<.0001
		AOM^*e*^	3.76	3.74, 3.78	

^*a*^Laboratory: one of the participating laboratories, the National Center for Lot Release, Green Cross Corp. (Korea), SK Chemicals (Korea), and Korea Vaccine Co. (Korea) (in randomized order)

^*b*^ *GMT*, Geometric mean titer

^*c*^ *95 % C.I.*, 95 % confidence interval

^*d*^ *PSM*, Plaque staining method

^*d*^ *AOM*, Agar overlay method

^*^ *P*-value: ANOVA test

Collaborative studies on the Korean reference material (code: 08/027) for the varicella vaccine began in 2009 [11]. Three laboratories participated every year, and another laboratory was added after 2012, bringing the total number of participating laboratories to four. To investigate the differences in test results between two methods, and to compare titers determined at the time of first manufacture and at the fifth year of manufacturing, we compared test method-based, year-based, and overall data, as described below. Table 3 shows a comparison of the virus content test methods for each year, which falls within an acceptance range of ± 0.5 log_10_PFU/ dose for each assigned titer. Comparison of the data collected in 2009 and 2012 showed that the difference between the test methods was 0.24 log_10_PFU/ dose (95 % confidence interval [CI], 0.18–0.30), which was within the acceptance range (−0.5–0.5 log_10_PFU/ dose), suggesting that the results of the two test methods were not significantly different. Analysis of the data collected in 2013 showed that the difference between the test methods was 0.19 log_10_PFU/ dose and that the 95 % CI (0.07–0.30) was within the acceptance range, further confirming that the results of the two methods were not significantly different. The differences between the data obtained in different implementation years were 0.04 log_10_PFU/ dose (95 % CI, −0.01–0.09) and −0.01 log_10_PFU/ dose (95 % CI, −0.15–0.12) for the PSM and AOM, respectively. These values were within the acceptance range ( −0.5–0.5 log_10_PFU/ dose), indicating that the results obtained in different implementation years did not differ significantly (Table 3). For the overall data, the 95 % CI was within the acceptance range (0.22 log_10_PFU/ dose; 95 % CI, 0.17–0.27), confirming that the results obtained using the two methods did not differ significantly (Table 4).

**Table 3:** Results of the collaborative study using the national standard (National Institute of Food & Drug Safety Evaluation, code no. 08/027) by year and method. The results of the virus content testing were compared to the test results of each collaborative study during 2009 ~ 2013 (titer unit: log_10_PFU/ dose)

Method	Experiment	Result		
		N^*d*^	Mean^*e*^ ± SD^*f*^	95 % CI^*g*^
PSM^*a*^	CSV^*c*^ 2012	41	4.50 ± 0.14	−0.01, 0.09
	CSV^*c*^ 2013	40	4.46 ± 0.14	
AOM^*b*^	CSV^*c*^ 2009	25	4.26 ± 0.09	−0.15, 0.12
	CSV^*c*^ 2013	40	4.27 ± 0.34	

^*a*^ *PSM*, Plaque staining method

^*b*^ *AOM*, Agar overlay method

^*c*^ *CVA*, Collaborative study for Varicella national standard material

^*d*^ *N*, Number of tests

^*e*^ *Mean,* Geometric mean titer

^*f*^ *SD*, Standard deviation

^*g*^ *CI*, confidence interval

**Table 4:** Analysis of the overall data from the collaborative study. The results of the collaborative study by method were compared to the virus content (titer) of the national standard (National Institute of Food & Drug Safety Evaluation, code no. 08/027) (titer unit: log_10_PFU/dose)

Method	Result		
	N^*c*^	Mean^*d*^ ± SD^*e*^	95 % CI^*f*^
PSM^*a*^	81	4.50 ± 0.14	0.17, 0.27
AOM^*b*^	65	4.27 ± 0.26	

^*a*^ *PSM*, Plaque staining method

^*b*^ *AOM*, Agar overlay method

^*c*^ *N*, Number of tests

^*d*^ *Mean*, Geometric mean titer

^*e*^ *SD*, Standard deviation

^*f*^ *CI*, Confidence interval

## Discussion

The Korean reference material was established by a previous collaborative study, which included the manufacturers [11]. However, in products that use the Oka strain, the virus content is usually measured by the PSM. For national lot release testing, quality control of all domestic products is performed using the AOM and PSM before human use. As such, we conducted this study to analyze varicella vaccines developed using different strains of virus with the aim of developing an accurate and reproducible test method, confirming its feasibility, and standardizing the method and reference material used for lot release testing.

The use of the PSM could shorten the test period from 11 days, which is required for the current AOM, to 9 days. Moreover, the use of the PSM could also reduce the workload of laboratory technicians secondary and tertiary overlays are omitted, and cell fixation and staining can be performed simultaneously. In addition, the plaques formed using the PSM staining method are more vivid and clear than those formed using the AOM, yielding more objective and accurate results. In this study, we showed that virus content testing performed using standardized methods and the same reference material will increase the reliability of the results, and lead to more consistent quality control of vaccine products.

## Conclusion

Varicella infections typically occur in children, mainly due to primary infection by the varicella virus. In contrast, shingles occurs in elderly or immune-compromised individuals due to activation of the latent virus [12]. Currently, domestic pharmaceutical companies and foreign companies, including GlaxoSmithKline (Belgium) and Merck (USA), are actively performing studies to develop varicella and shingles vaccines [13, 14]. The data presented in our current study will help to provide more accurate and reliable quality control measures for the current vaccine market. In addition, these results may lead to advancement in studies on various novel products, including recombinant varicella vaccines.

## Abbreviations

NCLR: National Center for Lot Release
AOM: Agar overlay method
PSM: Plaque staining
KRMVV: Korean reference material for the varicella vaccine
CPE: Cytopathic effect
ECACC: European Collection of Cell Cultures
PDL: Population doubling levels
IQR: Normalized interquartile range
CV: Coefficient of variation
GMT: Geometric mean titer
GCV: Geometric coefficient of variation
ACV: arithmetic coefficient of variation
